# Suspected *Pasteurella* Infection Associated With Catnip Exposure Leading to Finger Extensor Tendon Rupture: A Case Report

**DOI:** 10.1155/crdi/2619502

**Published:** 2026-10-28

**Authors:** Kennedy L. Couch, Holly C. Heck, Amar Ifetiha, Chloe Kin, William A. Kerr, Abdulazim Mustapha, Kate Szymanski, Kathryn M. Eisenmann

**Affiliations:** ^1^ Department of Medical Education College of Medicine and Life Sciences University of Toledo 3000 Arlington Ave, Mail Stop 1050 Toledo 43614 Ohio USA utoledo.edu; ^2^ Department of Orthopedics College of Medicine and Life Sciences University of Toledo 3000 Arlington Ave, Mail Stop 1010 Toledo 43614 Ohio USA utoledo.edu; ^3^ Department of Family Medicine College of Medicine and Life Sciences University of Toledo 3000 Arlington Ave, Mail Stop 1010 Toledo 43614 Ohio USA utoledo.edu

**Keywords:** case report, catnip exposure, extensor tendon rupture, hand infection, *Pasteurella multocida*

## Abstract

*Pasteurella* species commonly cause soft tissue infections following direct animal bites or scratches. However, transmission through indirect contact with contaminated materials remains underrecognized. We report an unusual, suspected case of *Pasteurella multocida* infection acquired through contact with catnip contaminated by feline saliva, resulting in progressive soft tissue infection and extensor tendon rupture in the hand of an otherwise healthy individual. Operative debridement, tendon repair, culture‐directed antibiotic therapy, and structured occupational therapy resulted in functional recovery with grip strength equal to the uninjured hand, a residual 37° total extension deficit, and a Quick Disabilities of the Arm, Shoulder, and Hand (QuickDASH) score of 4.55% at four months postinjury. This case emphasizes the importance of recognizing atypical transmission routes and implementing timely interventions to prevent severe complications.

## 1. Introduction

*Pasteurella multocida* is a facultative anaerobic, Gram‐negative coccobacillus that colonizes the oral flora of cats and dogs [1]. While well‐established as a cause of cellulitis, abscess formation, and deeper musculoskeletal infections following direct animal contact, indirect transmission mechanisms remain poorly understood [2]. We present a case of *Pasteurella* infection suspected to be contracted through exposure to catnip plant material contaminated with feline saliva, ultimately leading to extensor tendon rupture of the right long finger (RLF).

## 2. Case Presentation

A 51‐year‐old right‐handed female presented to her primary care provider (PCP) on Friday, January 17, 2025, one day after falling on ice in her driveway (Thursday, January 16, 2025). Her medical history was notable for no immunocompromising conditions, diabetes, asplenia, liver disease, or prior MRSA infection, and she was not taking immunosuppressive medications. She had no known drug allergies. She was a former light smoker who quit approximately 30 years earlier and reported minimal alcohol use. Family history was noncontributory. She worked in a nonclinical academic setting, lived in a rural/residential area, and maintained a home garden that included catnip plants. She reported falling backward into the adjacent garden bed while taking out the trash and landing on her right hand and wrist. Upon examination, there was mild erythema and swelling, specifically at the base of the RLF metacarpophalangeal (MCP) joint (Figure 1A). Small plant material was also removed by flushing. Radiographs of the right hand and wrist showed no fracture. No radiopaque foreign material was identified on subsequent review of these images by the orthopedic team.

**FIGURE 1:**
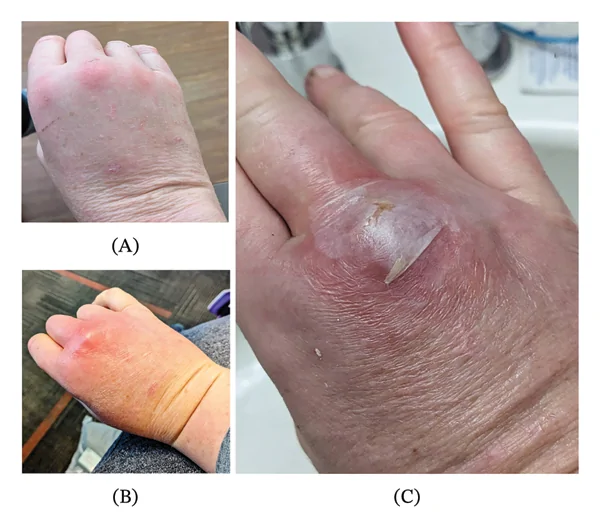
Initial presentation of patient to clinic. (A) Day 1 postinjury and initial presentation to the PCP. (B) Day 4 postinjury with abscess formation and swelling. (C) Day 7 postinjury and follow‐up with PCP.

Between January 17 and January 20, the patient was traveling out of state. Due to worsening inflammation, swelling, and development of a small abscess at the site of the injury (Figure 1B), the patient presented to an out‐of‐state urgent care on Monday, January 20, 2025, where she was prescribed sulfamethoxazole/trimethoprim (Bactrim) 800–160 mg tablet twice daily for 7 days empirically, consistent with MRSA‐directed therapy for purulent skin and soft tissue infections [3, 4]. The patient was advised to apply warm compresses.

Upon returning from travel on January 20, and in response to continually worsening inflammation, abscess development, and peeling skin, the patient followed up with her PCP on January 23, 2025, and a fluctuant area consistent with an abscess was noted (Figure 1C). During the patient interview, the patient mentioned that she believed she landed in a catnip plant frequented by free‐roaming neighborhood cats upon her fall.

Given the proximity of the wound to the joint, the PCP contacted the hand surgery team to expedite a previously scheduled follow‐up of February 13, 2025, to January 29, 2025. The orthopedic team approved an in‐office incision and drainage (I&D), which the PCP performed at that January 23 visit (Figure 2A). A multiloculated abscess was debrided, and the wound was packed with iodoform gauze. Aerobic and anaerobic wound cultures were obtained at the time of the I&D. Antibiotics were changed by the PCP from Bactrim to doxycycline (vibramycin) 100 mg capsule, twice daily for 10 days; amoxicillin–clavulanate (Augmentin) 875–125 mg tablet twice daily for 10 days for broader bacterial coverage, including *Pasteurella*. Fluconazole (Diflucan) 150 mg was also prescribed as a single dose, to be repeated in 7 days if symptoms persisted, to cover possible fungal infection from plant material inoculation and postantibiotic candidiasis, while awaiting wound culture results. Documentation of the wound is shown in Figure 2B,C.

**FIGURE 2:**
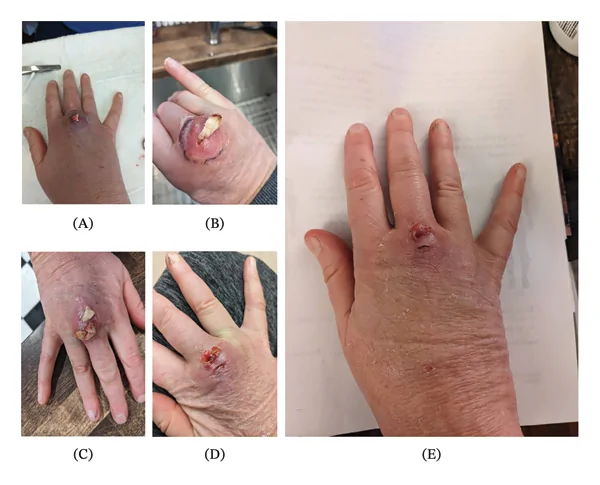
Follow‐up with PCP and in‐office incision and drainage. (A) Day 7 postinjury and post in office I&D procedure. (B) Day 8 postinjury. (C) Day 12 postinjury. (D) Day 13, postinjury, and packing removed. (E) Day 15 postinjury.

Several factors complicated early diagnosis. At the time of the urgent care visit, the patient was traveling out of state, which delayed reassessment by the PCP. The urgent care visit took place outside the patient’s primary health system, and its record was not accessible to the treating team. No culture was obtained, and no drainage was performed at the urgent care visit; the first drainage and cultures were performed on Day 7. The exposure history linking the injury to a catnip plant frequented by free‐roaming cats was not elicited until the Day‐7 visit. The plant material was not tested, and susceptibility testing of the isolate was not available.

Upon initial visit with orthopedics on January 29, 2025 (Day 13 postinjury, Figure 2D,E), the patient had a decreased ability to extend her RLF, with an extensor lag of approximately 30°, and wound cultures indicated the presence of *Pasteurella multocida* (aerobic culture finalized January 25, 2025 [Day 9], reported as rare growth, with a Gram stain showing few polymorphonuclear leukocytes and no organisms seen; no anaerobes were isolated). Due to the elective nature of the surgery and the infection seemingly being suppressed, orthopedics recommended an operative I&D. This was scheduled for February 4, 2025 (Day 19 postinjury), and the patient was instructed to continue both Augmentin and doxycycline until the surgery. In the judgment of the operating surgeon, the 30° extensor lag at consultation already indicated complete disruption of the extensor mechanism, and the interval to surgery was not considered to have contributed to further attritional tearing of the tendon.

Given the patient’s progressive loss of extension in the RLF, clinical evidence of joint violation, and a wound culture positive for *Pasteurella multocida*, operative intervention was clearly indicated. The goals of surgery were to achieve source control, debride infected and necrotic tissue, restore tendon integrity, and prevent further joint or systemic complications [5]. *Pasteurella multocida* is known to cause rapid tissue destruction, septic arthritis, and tenosynovitis when left untreated, particularly in deep or periarticular infections [6, 7].

Under monitored anesthesia care, the patient’s right arm was prepped and draped in a standard sterile fashion. The procedure was performed without tourniquet control. A solution of 0.5% Marcaine, and 1% lidocaine was infiltrated at the level of the long finger MCP region.

An elliptical incision was made over the dorsal MCP joint, incorporating a 0.5 × 1.0 cm area of compromised granulation tissue. Sharp dissection and rongeur debridement revealed complete transection of the extensor tendon and full‐thickness disruption of the joint capsule. There was no purulent fluid, and healed granulation tissue overlay the dorsal MCP joint. The joint space was clearly violated, raising concern for potential septic arthritis if left untreated.

The wound and joint were irrigated with 1 L of sterile normal saline delivered via a cystoscopy tubing technique.

Following thorough irrigation and debridement, the extensor tendon was repaired with a 2‐0 Ethibond suture in a locking horizontal mattress fashion to provide strong tension‐holding capacity and alignment. Skin was reirrigated and closed with 4‐0 Prolene in a horizontal mattress technique to reduce skin edge tension. A sterile dressing was applied, and the digit was immobilized in a volar resting splint with the MCP joint in slight flexion to minimize stress on the repair and optimize healing biomechanics (Figure 3A,B). This positioning is consistent with the management principles of Zone V extensor tendon injuries [8].

**FIGURE 3:**
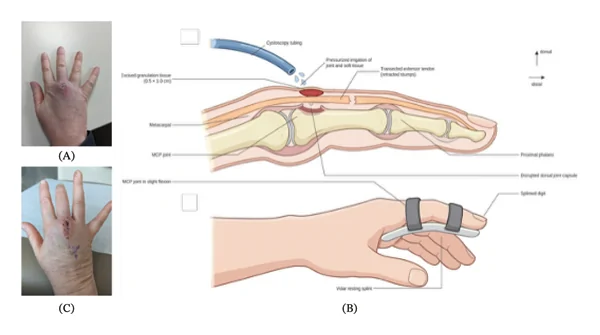
Pre‐ and postoperative surgical repair. (A) Day 19 photograph postinjury, preoperative. (B) Sagittal illustrated schematic of the right long finger at the metacarpophalangeal (MCP) joint; dorsal is up and distal is to the right. A 0.5 × 1.0 cm area of compromised granulation tissue was excised through a dorsal elliptical incision over the joint. The extensor tendon is completely transected, with retracted proximal and distal stumps and an intervening gap; the dorsal joint capsule is disrupted, while the volar capsule remains intact. The joint and surrounding soft tissue were irrigated with 1 L of sterile normal saline delivered through cystoscopy tubing. The tendon is shown before repairing; it was subsequently repaired with 2‐0 ethibond in a locking horizontal mattress configuration. The digit was immobilized postoperatively in a volar resting splint with the MCP joint in slight flexion (lower panel, shown on a representative digit). Schematic created by the authors with assistance from licensed FigureLabs software, not to scale. (C) Day 29 photograph postinjury, 10 days postoperative view of hand.

Postoperatively, the patient was maintained on oral doxycycline and amoxicillin–clavulanate for 3 additional days, based on wound culture results and Infectious Diseases Society of America (IDSA) guidelines for polymicrobial infections involving *Pasteurella* [5]. The patient was scheduled for follow‐up in the hand surgery clinic for wound checks, suture removal (Figure 3C), and initiation of hand therapy to restore range of motion and function relative to the preinjury and the uninjured hand (Figure 4A,B). A custom RLF relative motion orthosis was fabricated on Postoperative Day 10, ensuring good fit and mobility of all allowed joints (Figure 4C). The RLF was positioned in the recommended 20°–30° more extension than the neighboring digits while still allowing flexion of all other digits, with only RLF MP joint limitation at end‐range flexion. At the orthopedic assessment ∼7 weeks after incision, debridement, and repair (Day 70 postinjury), the RLF reached full extension on clinical examination, with MCP motion of 0°–65°. The patient was referred for hand therapy at that visit and completed eight sessions of occupational therapy between April 1 and May 15, 2025. At the occupational therapy evaluation on Day 75, goniometry recorded a 16° extension lag at the MCP joint and a 30° lag at the proximal interphalangeal (PIP) joint, with grip strength averaging 52.7 lbs on the right and 65.0 lbs on the left. Over the therapy course, PIP extension improved to a 15° lag while the MCP lag measured 22° at discharge on Day 119 postinjury, neither limited function. Right grip strength rose to a mean of 65.0 lbs across three trials, equal to the uninjured contralateral hand, which was unchanged over the same interval, and the Quick Disabilities of the Arm, Shoulder, and Hand (QuickDASH) score improved from 29.55% to 4.55%. Comparison with published outcomes after extensor tendon repair in the proximal zones is mixed. The total extension deficit of 37° corresponds to a fair result by Miller’s criteria [9] and exceeds both the mean final extension deficit of 6.7° reported across 86 zone V–VIII digits [10] and the 0°–8° lag reported at 12 weeks under early active mobilization protocols [11]. This is consistent with the dorsal MCP scar adhesion documented and treated throughout the therapy course. QuickDASH was not reported in either series, so the 4.55% score is presented without a direct comparator. The patient reported no pain at rest, with mild pain on gripping, mild tingling, and mild difficulty opening a tight jar as the residual symptoms and was discharged with no functional limitations and a home program of scar massage and resistive exercise to gain further MCP extension (Figure 4D). The clinical timeline from injury through discharge is summarized in Figure 5.

**FIGURE 4:**
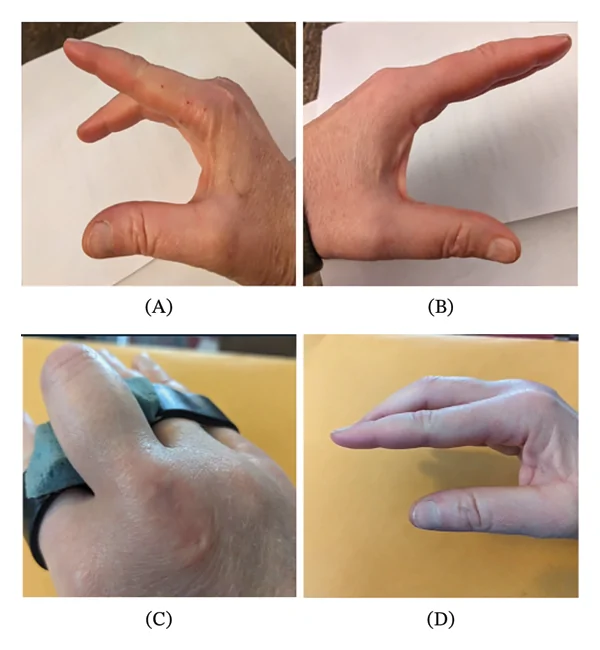
Pre‐ and postoperative recovery of extension. (A) right (affected) and (B) left hand (unaffected) preoperatively, Day 19 postinjury. (C) Postoperative and Day 60 postinjury, splinted and (D) without splint showing both improved wound healing and extension.

**FIGURE 5:**
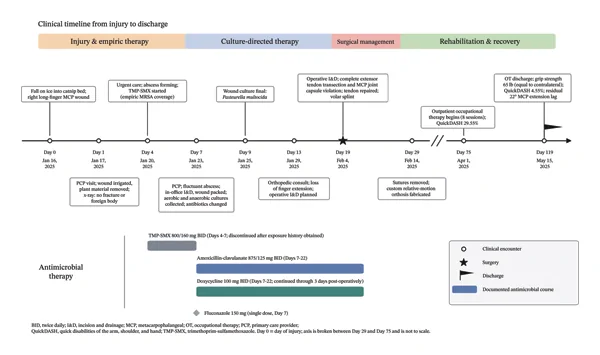
Clinical timeline from injury to discharge. Encounters, diagnostic results, and interventions are shown relative to the day of injury (Day 0, january 16, 2025), with calendar dates below each time point. Phase bands above the axis group the course into injury and empiric therapy, culture‐directed therapy, surgical management, and rehabilitation. The wound culture obtained at the Day‐7 incision and drainage grew *Pasteurella multocida* (finalized Day 9); no anaerobes were isolated. The lower panel shows the antimicrobial course, including the change from empiric trimethoprim–sulfamethoxazole to amoxicillin–clavulanate plus doxycycline after the animal‐associated exposure was identified. At occupational therapy discharge (Day 119), grip strength equaled the contralateral hand, and the QuickDASH score was 4.55%, with a residual 22° MCP extension lag. The time axis is broken between Day 29 and Day 75 and is not to scale. BID, twice daily; I&D, incision and drainage; MCP, metacarpophalangeal; OT, occupational therapy; PCP, primary care provider; QuickDASH, quick disabilities of the arm, shoulder, and hand; TMP‐SMX, trimethoprim–sulfamethoxazole. Figure created by the authors with assistance from licensed FigureLabs software.

## 3. Discussion

*Pasteurella* infections from nonbite exposures are uncommon but clinically significant [2]. *Pasteurella* typically colonizes the upper airway in healthy pets, farm animals, and various wild animals [7]. Transmission is typically the result of biting, scratching, licking, or nasopharyngeal secretions, but a particular case series of 79 cases included 34 of nonbite etiology. These mechanisms of infection included stepping in dog drool with an open submetatarsal ulcer, cat hair, abrasions after stumbling over a dog, and consuming half eaten food shared with a dog [2]. There have even been reported cases of vertical transmission as well as cases of *Pasteurella* epiglottitis [12, 13].

*Pasteurella* is typically known to spread to humans through pet bites or scratches, but there are many reported cases of transmission without evidence of either [14]. This is one such case in the literature involving a dog‐owner who developed an epicardial abscess containing *P. multocida* without any underlying bite or scratch [15]. Another case includes an elderly, immunocompetent cat‐owner that developed sepsis as well as septic arthritis due to *P. multocida* [16]. Yet another case illustrates an elderly cat owner that developed an ultimately fatal necrotizing fasciitis despite a lack of cutaneous findings [17]. These three cases demonstrate that the absence of trauma does not disqualify *P. multocida* as a possible source of infection.

Transmission through contaminated surfaces, such as catnip, plants, or toys, should be considered in patients presenting with soft tissue infections. The pathogenesis likely involves bacterial inoculation through microscopic skin breaches, leading to rapid progression due to the virulence of *P. multocida* [6]. *Pasteurella* transmission typically involves direct animal contact, but a biologically plausible mechanism of indirect transmission includes environmental survival of the bacteria through animal secretions [18, 19]. In this case, a catnip plant provided a plausible route for indirect inoculation through soft tissue trauma, although evidence of environmental inoculation was not directly demonstrated because the plant material was not tested at the time of injury.

This case also emphasizes the potential for deep tissue involvement, including tendon rupture, underscoring the necessity of early surgical intervention in severe infections. Methicillin‐resistant *Staphylococcus aureus* (MRSA) is an important and commonly isolated pathogen in community‐acquired hand infections [20, 21]. However, one study found that 6%–11% of septic arthritis cases in the hand were caused by *P. multocida* [22]. While Gram‐positive organisms such as *S. aureus* account for most soft tissue infections, *P. multocida* can progress rapidly, with signs often appearing within 3–48 h of inoculation [6, 23]. This case highlights the importance of considering *P. multocida* in atypical exposure scenarios.

On Day 4, the urgent care clinician prescribed trimethoprim‐sulfamethoxazole empirically for the purulent hand infection. This was a reasonable choice based on the clinical information available at the time, given the importance of MRSA as a cause of community‐acquired hand infections [5, 21]. Although plant material had been removed from the wound at the initial visit, the history of linking the exposure to a catnip plant frequented by free‐roaming cats was not elicited until Day 7. Trimethoprim‐sulfamethoxazole has activity against *P. multocida* [5, 24], but susceptibility testing of the patient’s isolate was not available, so the continued progression cannot be attributed to antimicrobial resistance. The abscess was not drained at the urgent care visit. Because I&D are the primary treatment for cutaneous abscesses, inadequate source control was a potential contributor to progression [5]. At the Day‐7 visit, the fluctuant abscess was drained, aerobic and anaerobic cultures were obtained, and therapy was broadened to amoxicillin–clavulanate plus doxycycline considering the newly identified suspected animal‐associated exposure. For similar presentations, we recommend (1) asking specifically about animal contact and about plants, soil, or surfaces frequented by animals; (2) draining fluctuant collections promptly and obtaining cultures at the first drainage procedure; (3) when an animal‐associated exposure is suspected, choosing empiric therapy active against *Pasteurella* and other expected organisms, with amoxicillin–clavulanate as an established first‐line option, and avoiding cephalexin, dicloxacillin, or erythromycin as sole therapy [5, 6]; and (4) considering early hand‐surgery consultation for wounds near a joint or with loss of tendon function.

Each year animal bites comprise 1% of ED visits; dog bites make up most of these cases, 85%–90%, with cat bites being a noteworthy 5%–10% [25]. When providing care for patients in contact with animals, it is also important to consider other common zoonotic infections. Animal bites produce a broad differential including *Staphylococcus*, *Streptococcus*, *Capnocytophaga*, *Bartonella*, or other anaerobic bacteria [25–27]. *Pasteurella* is a fast‐growing bacterium with infections often presenting within 3–48 h of an animal bite which can help differentiate from others which present later [6, 23].

*P. multocida* has the potential to cause complications including osteomyelitis, tenosynovitis, and septic arthritis [20]. Although rare, osteomyelitis infections require surgical debridement. Tenosynovitis is also an important possibility to consider which involves inflammation of the sheath surrounding the tendon. Tenosynovitis is more common in the flexor tendons and has the possibility to negatively impact postoperative range of motion after debridement, especially for delayed cases [28]. To avoid more serious complications and possible surgeries, it is important to quickly identify and treat suspected microorganisms. In this patient’s case, I&D prevented further complications, and the patient went on to make a functional recovery, with no pain at rest or difficulties with day‐to‐day tasks.

This case has several limitations. The transmission route remains presumptive, as environmental cultures of the catnip plant material were not obtained at the time of injury, and feline saliva contamination could not be directly confirmed. The association between the catnip exposure and *Pasteurella multocida* infection is based on the patient’s clinical history, the presence of plant material in the wound, and the known colonization of feline oral flora by *P. multocida* [1, 2]. Additionally, this is a single case report, and the generalizability of these findings is inherently limited. The urgent care record from Day 4 was not available, so the reasoning behind the initial empiric trimethoprim–sulfamethoxazole could not be reviewed. Susceptibility testing of the *P. multocida* isolate was also unavailable; therefore, resistance to the initial agent cannot be excluded. The wound culture was reported as rare growth with no organisms seen on Gram stain, indicating a low organism burden at the time of drainage. The contribution of antimicrobial selection versus delayed source control to the progression of infection cannot be determined from this single case. Finally, residual extension lag at the MCP and PIP joints was present at discharge, although measured function was unaffected. Strengths of this report include culture‐confirmed identification of the causative organism, detailed documentation of the clinical timeline from injury through operative management, and successful functional outcome following surgical repair.

## 4. Conclusion

This case illustrates an unusual route of *Pasteurella* infection, leading to severe musculoskeletal complications. Indirect exposure from contaminated plant material should be more widely recognized as a potential risk factor. Prompt diagnosis, appropriate antimicrobial therapy, and timely surgical management are essential for preventing long‐term morbidity in affected patients [29]. Possible complications if left untreated include osteomyelitis, tenosynovitis, and septic arthritis which can lead to the need for surgical interventions including debridement, tendon reconstruction, and potential amputation. *P. multocida* also makes up a smaller percentage of hand infections compared to its Gram‐positive counterparts, which means that swift identification is needed to properly treat an infection. Thankfully, *P. multocida* is sensitive to a broad spectrum of antibiotics. Increased awareness among clinicians may aid in the early identification and management of such atypical infections, especially in areas with higher free‐roaming cat populations.

## Author Contributions

Kennedy L. Couch, Holly C. Heck, Amar Ifetiha, Chloe Kin, and William A. Kerr contributed to the conceptualization of the case report, data collection, literature review, and drafting of the initial manuscript. Abdulazim Mustapha, Kate Szymanski, and Kathryn M. Eisenmann were involved in clinical oversight and data interpretation. Kathryn M. Eisenmann supervised the project.

## Funding

Funding for this case report was from internal departmental funds from the University of Toledo College of Medicine and Life Sciences Department of Medical Education.

## Disclosure

All authors revised the manuscript for intellectual content, approved the final version for submission, and agreed to be accountable for the work.

## Consent

Written informed consent was obtained from the patient for publication of this case report and accompanying images. This study was exempt from IRB approval.

## Conflicts of Interest

The authors declare no conflicts of interest.

## Data Availability

All data underlying the results are presented within the published article and its figures.

## References

[1] Peng Z. , Wang X. , Zhou R. , Chen H. , Wilson B. A. , and Wu B. , *Pasteurella multocida*: Genotypes and Genomics, Microbiology and Molecular Biology Reviews. (2019) 83, no. 4, e00014–e00019, 10.1128/mmbr.00014-19. PMC6759666 31484691

[2] Kannangara D. W. , Pandya D. , and Patel P. , *Pasteurella multocida* Infections With Unusual Modes of Transmission From Animals to Humans: A Study of 79 Cases With 34 Nonbite Transmissions, Vector Borne and Zoonotic Diseases. (2020) 20, no. 9, 637–651, 10.1089/vbz.2019.2558. 32423307

[3] Liu C. , Bayer A. , Cosgrove S. E. et al., Clinical Practice Guidelines by the Infectious Diseases Society of America for the Treatment of Methicillin-Resistant *Staphylococcus aureus* Infections in Adults and Children, Clinical Infectious Diseases. (2011) 52, no. 3, e18–e55, 10.1093/cid/ciq146. 21208910

[4] Talan D. A. , Mower W. R. , Krishnadasan A. et al., Trimethoprim-Sulfamethoxazole Versus Placebo for Uncomplicated Skin Abscess, New England Journal of Medicine. (2016) 374, no. 9, 823–832, 10.1056/nejmoa1507476. PMC4851110 26962903

[5] Stevens D. L. , Bisno A. L. , Chambers H. F. et al., Practice Guidelines for the Diagnosis and Management of Skin and Soft Tissue Infections: 2014 Update by the Infectious Diseases Society of America, Clinical Infectious Diseases. (2014) 59, no. 2, e10–e52, 10.1093/cid/ciu296. 24973422

[6] Hasan J. and Hug M. , Pasteurella multocida, 2023, StatPearls. 32491561

[7] Piorunek M. , Brajer-Luftmann B. , and Walkowiak J. , *Pasteurella multocida* Infection in Humans, Pathogens. (2023) 12, no. 10, 10.3390/pathogens12101210. PMC10610061 37887726

[8] Newport M. L. and Tucker R. L. , New Perspectives on Extensor Tendon Repair and Implications for Rehabilitation, Journal of Hand Therapy. (2005) 18, no. 2, 175–181, 10.1197/j.jht.2005.01.006. 15891975

[9] Miller H. , Repair of Severed Tendons of the Hand and Wrist: Statistical Analysis of 300 Cases, Surgery Gynecology & Obstetrics. (1942) 75, 693–698.

[10] Dalton S. S. , Maharjan L. M. , Yousuf H. , and Pientka W. F. , Extensor Tendon Repair Outcomes Based on Zone of Injury, Hand. (2024) 19, no. 5, 831–836, 10.1177/15589447221150510. PMC11284996 36734256

[11] Collocott S. J. F. , Kelly E. , and Ellis R. F. , Optimal Early Active Mobilisation Protocol After Extensor Tendon Repairs in Zones V and VI: a Systematic Review of Literature, Hand Therapy. (2018) 23, no. 1, 3–18, 10.1177/1758998317729713. PMC5846744 29593839

[12] Nakwan N. , Nakwan N. , Atta T. , and Chokephaibulkit K. , Neonatal Pasteurellosis: A Review of Reported Cases, Archives of Disease in Childhood—Fetal and Neonatal Edition. (2009) 94, no. 5, F373–F376, 10.1136/adc.2008.143982. 19208671

[13] Jan L. , Boute P. , and Mouawad F. , *Pasteurella multocida* Acute Epiglottitis, European Annals of Otorhinolaryngology, Head and Neck Diseases. (2021) 138, no. 2, 100–102, 10.1016/j.anorl.2020.06.008. 32600824

[14] Wei B. , Liu C. , Zhu J. , Zou X. , and Zhang Z. , *Pasteurella multocida* Infection: A Differential Retrospective Study of 482 Cases of *P. multocida* Infection in Patient of Different Ages, BMC Infectious Diseases. (2025) 25, no. 1, 10.1186/s12879-025-10711-1. PMC11881477 40038596

[15] McPherson D. F. , Menghani S. V. , Bhalodi R. G. , and Roesch T. A. , Canine Conundrum: Non-Bite Transmission Leading to *Pasteurella multocida* Epicardial Abscess, Americas Journal of Medicine. (2024) 137, no. 1, e3–e5, 10.1016/j.amjmed.2023.09.011. 37757962

[16] Kuwahara M. and Imanaka H. , *Pasteurella multocida* Bacteremia and Septic Arthritis Without an Animal Bite in an Older Patient Successfully Managed Without Joint Drainage: A Case Report, Cureus. (2026) 18, no. 2, 10.7759/cureus.102807. PMC12957895 41788112

[17] Vogt D. J. , Frey V. , Valek R. , and Buonomano R. , Fatal Necrotizing Fasciitis Caused by *Pasteurella multocida* Without Cutaneous Findings: When Pain is the Clue, Cureus. (2026) 18, no. 1, 10.7759/cureus.102296. PMC12933506 41755976

[18] Wilson B. A. and Ho M. , *Pasteurella multocida*: From Zoonosis to Cellular Microbiology, Clinical Microbiology Reviews. (2013) 26, no. 3, 631–655, 10.1128/cmr.00024-13. PMC3719492 23824375

[19] Cebra C. and Cebra M. , Pugh D. G. and Baird A. N. , Chapter 16—Diseases of the Hematologic, Immunologic, and Lymphatic Systems (Multisystem Diseases), Sheep and Goat Medicine, 2012, 2nd edition, W. B. Saunders: Saint Louis, 466–502.

[20] Rojas-Neira J. A. , Slullitel P. , Rossi L. , and Camino-Willhuber G. , Infections About the Hand, Orthopaedics and Trauma, 2024, Springer, Cham.

[21] Kistler J. M. , Thoder J. J. , and Ilyas A. M. , MRSA Incidence and Antibiotic Trends in Urban Hand Infections: A 10-Year Longitudinal Study, Hand. (2019) 14, no. 4, 449–454, 10.1177/1558944717750921. PMC6760093 29322874

[22] Fuchsjäger N. , Winterleitner H. , Krause R. , Feierl G. , and Koch H. , Susceptibility of Microorganisms Causing Acute Hand Infections, PLoS One. (2019) 14, no. 8, 10.1371/journal.pone.0220555. PMC6705788 31437151

[23] Morgan M. and Palmer J. , Dog Bites, BMJ. (2007) 334, no. 7590, 413–417, 10.1136/bmj.39105.659919.be. PMC1804160 17322257

[24] Bae H. G. et al., Antimicrobial Susceptibility of Human Pasteurella multocida Isolates in Korea, 2022–2025, 2026, Infect Chemother. 10.3947/ic.2026.0009 PMC13338684 42405545

[25] Ellis R. and Ellis C. , Dog and Cat Bites, American Family Physician. (2014) 90, no. 4, 239–243. 25250997

[26] Rabinowitz P. M. , Gordon Z. , and Odofin L. , Pet-Related Infections, American Family Physician. (2007) 76, no. 9, 1314–1322. 18019874

[27] Cossaboom C. M. , Wallace R. M. , and Behravesh C. B. , Halsey E. S. et al., Zoonotic Exposures: Bites, Scratches, and Other Hazards, CDC Yellow Book, 2025, Centers for Disease Control and Prevention (US), Atlanta (GA). 41818578

[28] El Atrouni W. , Birt M. , and Weihe R. , An Update on Suppurative Tenosynovitis, Current Infectious Disease Reports. (2024) 26, no. 1, 31–38, 10.1007/s11908-023-00826-4.

[29] Barsi Z. E. , Allen J. , and Meza A. , More Than a Case of Cellulitis: *Pasteurella multocida* Bacteremia, Cureus. (2023) 15, no. 3, 10.7759/cureus.36096. PMC10097429 37065280

